# Protegrin-1 and Analogues Against *Acinetobacter baumannii*: A Narrative Review

**DOI:** 10.3390/ph18030289

**Published:** 2025-02-20

**Authors:** Giulio Rizzetto, Edoardo De Simoni, Elisa Molinelli, Cecilia Busignani, Corrado Tagliati, Daisy Gambini, Annamaria Offidani, Oriana Simonetti

**Affiliations:** Clinic of Dermatology, Department of Clinical and Molecular Sciences, Polytechnic University of Marche, 60126 Ancona, Italy; grizzetto92@hotmail.com (G.R.); edodesimoni@hotmail.it (E.D.S.); molinelli.elisa@gmail.com (E.M.); cecilia.busignani@gmail.com (C.B.); corradotagliati@gmail.com (C.T.); daisy.gambini@libero.it (D.G.); a.offidani@ospedaliriuniti.marche.it (A.O.)

**Keywords:** protegrin-1, *A. baumannii*, combination therapy, protegrin-1 analogues

## Abstract

*A. baumannii* is recognised as an important etiologic agent for hospital infections and increases the risk of postoperative complications, worsening mortality and prolonging hospitalisation. Protegrin-1 (PG-1) is one of the most promising antimicrobial peptides (AMPs) in the literature, since its antimicrobial action covers a wide range of Gram-positive and Gram-negative bacteria, including *A. baumannii.* PG-1 represents a valid new therapeutic option for the treatment of *A. baumannii* multi-drug resistant infections, showing synergic activity with traditional antibiotics, such as colistin. However, its clinical use in humans still requires studies, especially considering the haemolytic risk. For this reason, the use of PG-1 analogues, such as PLP-3, HV2, CDP-1, and IB367, seems to be the most promising way for the clinical use of this class of AMPs.

## 1. Introduction

*A. baumannii* is a Gram-negative bacterium that is often responsible for healthcare-associated infections, consistently in intensive care units and for people with preexisting comorbidities. In fact, *A. baumannii* is recognised as an important etiologic agent for hospital pneumonia, sepsis, skin and soft tissue infections, and surgical wound infections. These increase the risk of postoperative complications, worsening mortality and prolonging hospitalisation [1,2]. In addition, *A. baumannii* is known for its ability to develop broad-spectrum antibiotic resistance, including β-lactams, aminoglycosides, and carbapenems, leading to the inefficacy of many conventional therapies [2,3]. Therefore, *A. baumannii* represents a major challenge for the healthcare system and the search for new therapeutic options is necessary to overcome its multiple antibiotic resistances. In this context, antimicrobial peptides (AMPs) arise as a new promising therapeutic choice.

In fact, AMPs are oligopeptides produced by multiple organisms, including humans, and are a component of the innate immune system. The spectrum of action of AMPs includes a wide variety of microorganisms, such as bacteria, fungi, and viruses. This is possible through several mechanisms of action, including disruption of bacterial membranes, inhibition of protein synthesis, and modulation of immune responses [4,5]. AMPs may therefore represent an interesting therapeutic option, especially to overcome antibiotic resistance, due to their broad-spectrum activity, low risk of inducing singular resistance, and synergistic ability with other antibiotics [6,7,8].

More specifically, protegrin-1 (PG-1), a β-hairpin cysteine-rich cathelicidin with two disulfide bridges (6–15 and 8–13), is one of the most promising AMPs in the literature, since its antimicrobial action covers a wide range of Gram-positive and Gram-negative bacteria, including *A. baumannii* [9,10,11]. PG-1 shows its antimicrobial effect through various mechanisms. These include insertion into bacterial membranes and the creation of stable pores in planar lipid bilayers, leading to loss of membrane polarisation and cell death. This mechanism leads to cell membrane disruption within minutes [12]. PG-1 and PG-1 analogues have been studied in the literature, and it was noticed that linear PG-1 analogues do not exhibit regular secondary structures, lacking disulfide bridges. This leads to reduced antimicrobial activity. In fact, the total number of positively charged residues and the amphiphilicity of β-hairpin were identified as factors affecting antibacterial activity [13].

PG-1 and PG-1 analogues may therefore represent a therapeutic option that can overcome the increasingly spreading phenomena of *A. baumannii* antibiotic resistance, especially rescuing the efficacy of conventional antibiotics. For these reasons, we decided to conduct a narrative review of the literature focusing on the efficacy of PG-1 against *A. baumannii*. The purpose of this review is not only to summarise the prior findings on PG-1 and analogues against *A. baumannii* but also to suggest new topical vehicle methods for further studies.

## 2. Materials and Methods

A narrative review of the literature was performed. Data were obtained using the PubMed database and searching for the following keywords, alone or in combination: “protegrin-1”, “PG-1”, “*A. baumannii*”, “protegrin”, “Gram-negative”, “protegrin-1 analogues”, and “antibiotics”.

Particular attention was paid to the combination of PG-1 with other conventional antibiotics in use. No time limits were considered for the inclusion of studies. We included the most recent studies present in the literature; however, considering the specificity of this research field, we decided to include studies without time limits, in order to obtain a more complete overview. On the other hand, only high-quality, peer-reviewed studies with experimental data conducted in vivo or in vitro on *A. baumannii* strains were included in this review. Case reports and studies with non-reproducible data were excluded.

Molecules derived from PG-1 that have not been tested on *A. baumannii* were reviewed only if tested on Gram-negative bacteria in vitro and/or in vivo. Data on the efficacy of PG-1 and analogues against other Gram-negative bacteria were included, considering the reported indicators of efficacy.

## 3. Results

### 3.1. PG-1 Against A. baumannii

A summary of the activity of PG-1 against *A. baumannii* is reported in Table 1. PG-1 activity was evaluated alone against *A. baumannii* in the study by Morroni et al. [14]. Strains of *A. baumannii* were isolated from surgical wounds, so they were good indicators of microbiological conditions in a hospital environment.

- In Vitro

15 strains were colistin susceptible and 4 were colistin resistant. The antimicrobial activity of PG-1 was evaluated by determination of minimum inhibitory concentration (MIC) and minimum bactericidal concentration (MBC) and time-kill. The effect of PG-1 on the biofilm produced by *A. baumannii* was also tested, as well as the possible formation of resistance with cultures at increasing PG-1 concentrations. PG-1 was effective against all *A. baumannii* strains, both colistin-sensitive and colistin-resistant, at concentrations between 2 and 8 μg/mL. In almost all strains, the MBC was identical to the MIC or was one dilution higher. The MIC50 and MIC90 were 4 and 8 μg/mL, respectively [14]. However, PG-1 showed no antibiofilm activity against *A. baumannii* strains, while no resistance formation to PG-1 was observed.

### 3.2. PG-1-like Peptide (PLP)-3 Against A. baumannii

PLP-3 is a synthetically derived PG-1 analogue, which has a bicyclic antiparallel β-sheet conformation. This is consistent for reducing the haemolytic risk of PG-1 in mammals [16]. PLP-3 was synthesised considering the main features of protegrins against Gram-negative bacteria. In particular, the β-hairpin conformation, stabilised by disulfide bonds and intrachain hydrogen bonds, was maintained. The polycationic structure was also very important, allowing the creation of electrostatic interactions with the phosphate groups of the Gram-negative membrane. Finally, the hydrophobic side chains located at the centre of the β-hairpin allowed the formation of an amphipathic structure. This was functional for the interaction with the hydrophobic portion of the Gram-negative lipid bilayer [16]. PLP-3 therefore combines the characteristics of PG-1, thanks to a highly constrained macrocyclic structure, eliminating the flexible tails present in PG-1 and connecting the antiparallel β-strands of the peptide in a head-to-tail fashion. This more rigid structure allows greater antimicrobial potency and lower toxicity compared to natural protegrins.

PLP-3 is designed to enhance antimicrobial efficacy while maintaining stability against bacterial resistance mechanisms. In fact, PLP-3 exerts its antibacterial activity primarily through membrane disruption, similar to PG-1, by interacting with negatively charged bacterial membranes, leading to pore formation, membrane depolarisation, and subsequent bacterial cell lysis. This peptide demonstrates potent activity against multi-drug resistant (MDR) Gram-negative pathogens, including *E. coli*, *K. pneumoniae*, and *A. baumannii*, highlighting its broad-spectrum potential. Despite its high efficacy, bacteria may develop resistance through mechanisms such as membrane charge modifications (e.g., lipid A modification with phosphoethanolamine), efflux pump overexpression, or proteolytic degradation of the peptide by bacterial proteases. However, PLP-3 exhibits improved stability and reduced susceptibility to these resistance pathways.

- In Vitro

PLP-3 efficacy has been demonstrated in vitro against *A. baumannii* in several strains, including colistin-resistant strains and MDR strains. MIC90 was 2 mg/L for *A. baumannii* [16]. The haemolysis IC 50 value was 48.53 mg/L, while cytotoxicity against human cells showed values of ca. 200 mg/L. This consisted of a 100-fold selectivity window for bacterial over human cells [16]. This is very important, as PLP-3 seems much safer than PG-1 in terms of clinical application in humans.

In addition, the efficacy of PLP-3 was also tested against *P. aeruginosa* and *K. pneumoniae*, both susceptible and MDR strains. The PLP-3 MIC range was between 1 and 8 mg/L. More specifically, *P. aeruginosa* showed a higher MIC50 and MIC90 than *A. baumannii* (4 and 8 mg/L, respectively), showing an excellent bactericidal action for both colistin-resistant and MDR strains. On the other hand, considering *K. pneumoniae*, the MIC50 was 4 mg/L and the MIC90 was 16 mg/L, although the variability was higher than for the other bacteria considered (MIC50 from 2 to 32 mg/L).

### 3.3. PG-1 in Combination Against A. baumannii

- Colistin, In Vitro

Morroni et al. [14] showed that PG-1 in combination with colistin had synergistic action, with a fractional inhibitory concentration (FIC) < 0.5 for all strains of *A. baumannii*, including colistin-resistant strains. The time-kill assay also confirmed the synergic efficacy. However, there was no significant synergistic effect in this study with other antibiotics tested, such as fosfomycin, levofloxacin, meropenem, tigecycline, or rifampicin. No antagonistic effects were reported.

### 3.4. PG-1 and Analogues Against Gram-Negative Bacteria

- PG-1

PG-1 has also been used against other Gram-negative bacteria, showing excellent efficacy; however, its cytotoxicity has always limited its use. In this sense, numerous analogues have been analysed in the literature to exploit the antimicrobial action of PG-1, reducing its toxicity [17,18].

Amphiphilic β-hairpin antibacterial peptides, of which PG-1 is a typical representative, showed strong antimicrobial activity and may be an excellent stable scaffold for the development of peptide analogues [19]. For example, the replacement of lysine residues with histidine leads to reduced cytotoxicity, while maintaining antimicrobial efficacy with high cell selectivity [20,21].

- In Vitro

PG-1 showed interesting antibacterial activity against Gram-negatives; in particular, MIC values of 2 μM for *E. coli*, 4 μM for *S. pullorum*, and 8 μM for *P. aeruginosa* were reported in vitro [15].

- HV2

HV2, a PG-1-derived peptide with the structure RRVHVHVDPGVHVRR-NH2, is a histidine-rich β-hairpin-like AMP [15]. In this study, it was found that HV2 had excellent selectivity for Gram-negative bacteria. HV2 did not present its typical structure in an aqueous solution, but it was folded into the β-hairpin structure when interacting with trifluoroethyl alcohol. The implementation of HV2 from PG-1 was possible by action on the hydrophobic amino acids of different subtypes, influencing both the antibacterial activity and the cellular selectivity. In particular, HV2 had a significantly higher cellular selectivity than PG-1.

HV2 showed a detergent-like mechanism of action, with the dissolution of the bacterial cell membrane. More specifically, HV2 determined a highly permeabilised outer layer, depolarising the plasma membrane while permeabilising the inner membrane. This leads to membrane disruption, facilitated by electrostatic interactions between HV2 cationic residues and the negatively charged bacterial outer membrane. Upon binding, HV2 inserts into the lipid bilayer, leading to membrane permeabilisation, ion leakage, and eventual cell lysis. Additionally, HV2 can penetrate bacterial cells and interact with intracellular targets, further enhancing its bactericidal activity. This peptide demonstrates strong activity against drug-resistant Gram-negative pathogens, including *E. coli* and *P. aeruginosa*. Despite this efficacy, bacterial resistance to HV2 may arise through modifications of the outer membrane, such as lipid A remodelling to reduce peptide binding, efflux pump activation to expel the peptide, and enzymatic degradation by bacterial proteases. However, the structural stability and high membrane affinity of HV2 limit the development of resistance. Furthermore, HV2 was able to reduce the production of inflammatory factors thanks to its ability to bind to lipopolysaccharides [15].

- In Vitro

Considering in vitro studies, HV2 showed interesting antimicrobial action against Gram-negatives, particularly with MIC of 8 μM for *E. coli*, *S. pullorum*, and *P. aeruginosa*. On the other hand, it showed no action against Gram-positives, unlike PG-1, and its haemolytic activity was almost not detectable even at high concentrations. For example, at 128 μM, the haemolysis rate was less than 5%. This makes it an excellent candidate for clinical application, and it could also be effective against *A. baumannii*, although there are no specific studies on this.

- CDP-1

Cysteine-deleted protegrin-1 (CDP-1) is a PG-1 derivative that has a deletion for cysteines. In particular, Cys 6–Cys 15 and Cys 8–Cys 13 form stable disulphide bridges in PG-1, which appear to be responsive to antimicrobial action. CDP-1 has a broad-spectrum antibacterial action due to its structure as a Cys-deleted analogue of PG-1. In fact, CDP-1 interacts with negatively charged lipids such as lipopolysaccharides. The mechanism of action of CDP-1 consists of the disruption of the outer membrane with lipopolysaccharides, reaching through this permeability the inner plasma membrane [22]. CDP-1 folds into an amphipathic β-hairpin conformation after forming a complex with lipopolysaccharide micelles. This β-hairpin structure can insert itself between lipopolysaccharides, resulting in the loss of outer membrane packing in Gram-negative bacteria. The N-terminal sequence RGGR plays a crucial role in determining the formation of the β-hairpin structure in contact with lipopolysaccharides. However, a study by Mohanram et al. [22] demonstrated that CDP-1 has excellent antimicrobial action against Gram-negative bacteria by binding to the lipopolysaccharide of the outer membrane and then assuming a β-hairpin conformation. It exhibits strong antimicrobial activity against both Gram-negative and Gram-positive bacteria, including MDR strains. However, bacterial resistance to CDP-1 may arise through mechanisms such as LPS modification, increased expression of efflux pumps, and proteolytic degradation [22].

- In Vitro

In vitro investigations highlighted MIC for *E. coli* of 4 μM, *P. aeruginosa* of 8 μM, *K. Pneumoniae* of 8 μM, and *S. enterica* of 8 μM.

- Isaganan/IB367

Isaganan or IB367 is an analogue of PG-1 and is a 17-amino acid synthetic protegrin. The mechanism of action of IB367 is different between Gram-positive and negative bacteria. Considering Gram-negative, IB367 first interacts with LPS, neutralising its negative charge and allowing penetration into the outer membrane. Once inside, it reaches the inner membrane, where it integrates into the lipid bilayer and forms transmembrane pores [23]. These pores lead to uncontrolled ion exchange, leakage of cytoplasmic contents, and membrane depolarisation, ultimately causing cell death. For Gram-positive bacteria, IB367 directly interacts with the cytoplasmic membrane, targeting anionic phospholipids such as cardiolipin and phosphatidylglycerol. The peptide integrates into the bilayer, disrupting lipid packing and causing localised membrane thinning and pore formation, resulting in bacterial lysis [24]. Although IB367 has not been studied in the literature specifically against *A. baumannii*, it may also play an important role in the control of Gram-negative infections.

- In Vivo

In particular, IB367 was clinically used in a phase 3 study [25], applying it topically to the oral mucosa of 225 patients with mucositis from chemotherapy. IB367 was administered at a dosage of 9 mg/3 mL, six times daily for 21–28 days. A statistically significant reduction in the load of aerobic bacteria, streptococci, and yeast was observed at the end of therapy. In particular, Gram-negative bacilli were maintained at a detection level of less than 2 CFU/mL with a daily dosage of 54 mg. In a previous study, the reduction of oral Gram-negatives was retargeted to a higher dosage of 12 g/day [26].

- In Vitro

Another study, by Simonetti et al., reported the efficacy of IB367 [27]. More specifically, IB367 has been shown to be effective against *P. aeruginosa* and *E. coli*, alone and in combination with colistin and imipenem. In particular, in the in vitro model, IB367 showed similar efficacy to colistin and better efficacy than other conventional antibiotics such as imipenem, ceftazidime, piperacillin, ciprofloxacin, and amikacin. Synergistic action was then evidenced with IB367 and colistin or imipenem, with no antagonism with the other antibiotics considered. In an in vivo murine model on infected wounds, the efficacy of topical monotherapy of IB367 against *P. aeruginosa* and *E. coli* was shown, but the best result in terms of efficacy was observed with the combination of IB367 and colistin. Although no studies have been carried out on *A. baumannii*, these data are very promising for possible use on this pathogen as well.

- IR2

IR2 is a simplified symmetric-end peptide, synthesised by combining the β-turn of PG-1 with specific amino acid repeat sequences, including hydrophilic amino acids (R) and hydrophobic amino acids (I, F, W, and P). IR2, a peptide with 14 amino acid residues, showed a significantly higher level of cellular selectivity than PG-1. Unlike PG-1, IR2 was not structured in an aqueous solution but achieved a β-sheet and β-hairpin structure when interacting with a membrane environment [28].

IR2 kills bacterial cells by damaging the cell membrane, leading to cytosol leakage and cell lysis. Its mechanism of action is primarily based on an amphipathic structure that facilitates strong electrostatic and hydrophobic interactions with negatively charged bacterial membranes. Upon binding, IR2 rapidly integrates into the lipid bilayer, causing membrane perturbation and pore formation that leads to the leakage of essential intracellular contents and cell death.

- In Vitro

In vitro studies indicate that IR2 exhibits broad-spectrum antimicrobial activity against both Gram-negative and Gram-positive pathogens while displaying minimal cytotoxicity towards mammalian cells, and this may be explained through the selective membrane affinity of IR2. Moreover, because IR2’s bactericidal effect is largely based on physical disruption of the membrane rather than targeting specific intracellular processes, the likelihood of resistance development is reduced. Nevertheless, bacteria may still evolve resistance through modifications in membrane lipid composition or surface charge, which could decrease peptide binding efficiency. IR2 has an excellent profile in terms of both antibacterial action and reduced haemolysis. Its action in killing bacteria at MIC and 2-fold MIC was evaluated in an in vitro study. *E. coli* was chosen as a representative model for Gram-negative bacteria. In this case, 99.99% of *E. coli* were killed after 30 min of MIC exposure, while its action on Gram-positives would appear to be reduced. [28]. Again, there is a lack of specific data on *A. baumannii* but the action profile on Gram-negatives is interesting and makes it a promising study option.

A summary of reviewed studies about PG-1 analogues is reported in Table 2 and Table 3.

## 4. Discussion

The development of bacterial resistance towards conventional antibiotics is a major health concern, especially for MDR pathogens like *A. baumannii*, which is considered a priority considering the need for new treatments [29]. Combining AMPs with traditional antibiotics may enhance their antimicrobial efficacy, reduce the emergence of resistance, and improve therapeutic outcomes. This approach, known as antibiotic-peptide synergy, is gaining attention as a strategy to combat antibiotic-resistant infections.

Given the MDR profile of *A. baumannii*, our review of the literature reveals the synergistic utility of PG-1 and PG-1 analogues with conventional antibiotics, such as colistin, in the case of resistance development. This may be explained through the similar mechanisms of action of colistin and PG-1, with transmembrane pore formation leading to cell lysis, although PG-1 has different membrane targets. Specifically, PG-1 binds membrane phospholipids, leading to pore formation [12], while colistin binds lipid A of lipopolysaccharides [30]. Regarding the mechanism of action of PG-1, previous studies showed that PG-1 inserts itself into lipid membranes at an oblique angle, forming toroidal pores with induction of cell lysis [31,32,33]. Less significant results have been shown for the combination of PG-1 with fosfomycin, levofloxacin, meropenem, tigecycline, and rifampicin for *A. baumannii* [14]. However, the absence of antagonism phenomena makes it possible to use these antibiotics in combination. On the other hand, as reported in Table 4, the combination of meropenem and rifampicin was reported synergic with PG-1 against MDR Gram-positive in some studies [34,35,36].

In contrast, antibiotics such as fosfomycin, meropenem, and rifampicin showed no synergic effect in combination with PG-1 [14]. This could be explained since these antibiotics have different mechanisms that do not directly rely on or benefit from enhanced membrane permeability. Fosfomycin, for example, targets MurA in cell wall biosynthesis and requires specific transporters for uptake, while meropenem and rifampicin inhibit cell wall synthesis and RNA polymerase, respectively. Their efficacy is not significantly increased by the membrane disruption caused by PG-1, as their uptake or target engagement is governed by different bacterial processes. Additionally, resistance mechanisms to these antibiotics are typically mediated by enzymatic degradation, target modification, or altered intracellular uptake, which are not overcome by additional membrane permeabilisation [35].

Interestingly, the time-kill of *A. baumannii* was very effective at high PG-1 concentrations, i.e., 8 MICs, similar to other AMPs [37]. One element to be considered is the lack of antibiofilm action of PG-1, even at high doses. For this reason, the association with other AMPs acting as antibiofilm peptides might be useful [38]. One promising approach to enhance PG-1’s ability to penetrate and disrupt biofilms involves the deletion of cysteine residues, as seen in analogues like CDP-1, which removes disulfide bonds and increases the peptide’s structural flexibility. This may potentially allow a more effective diffusion through the dense extracellular polymeric substance of biofilms [22]. Other modifications include enhancing the net positive charge or amphipathicity of PG-1, thus improving the electrostatic and hydrophobic interactions with biofilm components. These strategies aim to overcome the limited antibiofilm activity of native PG-1 and improve its therapeutic potential against biofilm-associated infections [22].

On the other hand, PG-1 appears not to induce resistance phenomena in the various *A. baumannii* samples analysed, thus proposing itself as a potential antimicrobial lifesaver in MDR cases [14,39]. This is very important, considering that *A. baumannii* resistance is a cause of global concern. PG-1 exerts a rapid membrane-disrupting mechanism, and this may contribute to reduced resistance development compared to conventional antibiotics. Prolonged clinical use could still select for resistant bacterial phenotypes. Bacteria might adapt by modifying their cell envelope, for instance, altering the charge or composition of lipopolysaccharides in Gram-negative bacteria or the phospholipid content in Gram-positive bacteria, to decrease the electrostatic binding of PG-1 [35]. Additionally, overexpression of efflux pumps or the secretion of proteolytic enzymes capable of degrading the peptide could further compromise its efficacy. Thus, while PG-1 is promising as a potent antimicrobial agent, its long-term clinical application will require vigilant resistance monitoring and possibly combination therapies to mitigate the emergence of resistance [35].

The main obstacle to the wider application of PG-1 as an antibiotic lies in its mammalian toxicity, mainly due to its significant haemolytic impact on human red blood cells [40]. However, further studies are needed to evaluate possible safe uses in humans.

One solution is the use of PG-1 analogues such as PLP-3, which is effective against colistin-resistant strains of *A. baumannii* and currently represents the best option for clinical application [16]. Other PG-1 analogues have also shown significant efficacy against Gram-negatives, such as HV2, representing an interesting option for expanding research specifically against *A. baumannii*, with lower haemolytic risk compared to PG-1 [28,41].

On the other hand, the clinical application of PG-1 could be easier through topical application over surgical wounds infected with *A. baumannii*, or by exploiting liposomal vehicles that could limit the systemic spread of PG-1, similar to the case for other molecules such as LL-37 [42,43]. Another interesting solution for the clinical use of PG-1 may be the incorporation of PG-1 into a nanostructure by PEGylation of PG-1 terminals, reducing its cytotoxicity [44].

A final interesting aspect concerns the ability of PG-1 to regulate cell proliferation, particularly of pig granulosa cells. This occurs by increasing the expression of epidermal growth factor receptor (EGFR), and the phosphorylated-/total extracellular signal-regulated kinase (ERK)1/2 ratio [45]. Thus, PG-1 may also have roles in the regulation of growth factors of other cell types, such as cancer cells, or in chronic inflammatory conditions [46,47,48,49]. However, further studies are needed to investigate the role of the cellular regulation of PG-1.

## 5. Conclusions

PG-1 represents a valid new therapeutic option for the treatment of *A. baumannii* MDR infections, although its clinical use in humans still requires further studies, especially considering the haemolytic risk in mammals. For this reason, the use of PG-1 analogues, such as PLP-3, HV2, CDP-1, and IB367, seems to be the most promising clinical use of this class of AMPs. The possible topical vehiculation of PG-1, through liposomes or PEGylation, may represent a new promising approach for further investigation.

## Figures and Tables

**Table 1:** Summary of reviewed studies on PG-1.

Study	Molecule	Alone/Combination	Findings
Morroni et al. [14]	PG-1	Alone	- *A. baumannii* from surgical wounds, in vitro - PG-1 is effective at concentrations between 2 and 8 μg/mL - PG-1 showed no antibiofilm activity - No resistance formation was observed
		Colistin combination	- PG-1 plus colistin had a synergistic action, with in vitro fractional inhibitory concentration (FIC) < 0.5 for all strains of *A. baumannii*, including colistin-resistant strains - No synergistic effect with fosfomycin, levofloxacin, meropenem, tigecycline, or rifampicin - No antagonistic effects
Dong et al. [15]	PG-1	Alone	- Activity in vitro against Gram-negative: - MIC of 2 μM for *E. coli*, 4 μM for *S. pullorum*, and 8 μM for *P. aeruginosa*

PG-1: protegrin-1.

**Table 2:** Summary of reviewed studies on PG-1 analogues.

Study	Molecule	Alone/Combination	Findings
Moreno-Morales et al. [16]	PLP-3	Alone	- *A. baumannii* colistin-resistant and sensitive strains in vitro. - MIC90 was 2 mg/L for *A. baumannii.* - Cytotoxicity against human cells 200 mg/L. - 100-fold selectivity window for bacterial over human cells.
Dong et al. [15]	HV2	Alone	- In vitro MIC of 8 μM for *E. coli*, *S. pullorum*, and *P. aeruginosa*. - No action against Gram-positives. - At 128 μM, the haemolysis rate was less than 5%.
Mohanram et al. [22]	CDP-1	Alone	- Excellent antimicrobial action against Gram-negatives by binding to the lipopolysaccharide of the outer membrane and then assuming a β-hairpin conformation. - In vitro MIC for *E. coli* of 4 μM, *P. aeruginosa* of 8 μM, *K. pneumoniae* of 8 μM, and *S. enterica* of 8 μM.
Tamamura et al. [25]	Isaganan/IB367	Alone	- Phase 3 study topically applied to the oral mucosa of 225 patients with mucositis from chemotherapy. - 9 mg/3 mL, six times daily for 21–28 days. - Gram-negative bacilli less than 2 CFU/mL with a daily dosage of 54 mg.
Simonetti et al. [27]	Isaganan/IB367	Alone Colistin/Imipenem combination	- Effective against *P. aeruginosa* and *E. coli*, alone and in combination with colistin and imipenem. - An in vivo murine model had the best result for combination with colistin.
Dong et al. [28]	IR2	Alone	- 99.99% of *E. coli* were killed after 30 min of MIC exposure. - Gram-positives would appear to be reduced.

CDP-1: cysteine deleted protegrin–1; PG-1: protegrin-1; PLP-3: PG-1 like peptide 3.

**Table 3:** Comparison of PG-1 and PG-1 analogues.

	Antimicrobial Activity (Efficacy)	Toxicity	Clinical Applicability	
PG-1	Highly potent against both Gram-negative and Gram-positive bacteria with rapid bactericidal activity.	Exhibits dose-dependent cytotoxicity and haemolytic activity, which may limit its systemic use.	Primarily evaluated in preclinical studies; clinical translation is challenged by stability and toxicity issues.	Morroni et al. [14] Dong et al. [15]
CDP-1	Comparable antimicrobial activity to PG-1, particularly effective against Gram-negative bacteria.	Reduced toxicity relative to PG-1 due to improved selectivity resulting from the absence of disulfide-induced rigidity.	Considered a promising candidate for systemic applications; currently in preclinical evaluation.	Mohanram et al. [22]
IB367	Broad-spectrum activity is effective against multidrug-resistant (MDR) Gram-positive and Gram-negative bacteria.	Exhibits a favourable toxicity profile with minimal haemolytic effects, supporting its potential for clinical use.	Advanced clinical evaluation for topical applications (e.g., oral mucositis) with further development underway.	Tamamura et al. [25] Simonetti et al. [27]
PLP-3	Demonstrates potent activity against MDR Gram-negative pathogens in vitro.	Improved safety profile with reduced cytotoxicity compared to native PG-1.	Currently at the preclinical stage; shows promise for treating MDR infections.	Moreno-Morales et al. [16]
HV2	Exhibits potent bactericidal activity, particularly against Gram-negative bacteria such as *E. coli* and *Pseudomonas aeruginosa*.	Effective at bactericidal concentrations; potential toxicity requires optimisation to balance efficacy with minimal damage to mammalian cells.	Investigated in preclinical studies with promising in vitro efficacy.	Dong et al. [15]
IR2	High and selective antimicrobial potency against Gram-negative pathogens, with the potential for a broader spectrum due to its optimised design.	Enhanced selectivity with reduced toxicity relative to PG-1, making it attractive for therapeutic applications.	Currently in the preclinical stage with potential for targeting MDR infections.	Dong et al. [28]

**Table 4:** PG-1 and combination therapy for MDR pathogens.

Combination Agent	Mechanism of Synergy	Target MDR Pathogens	Observed Outcome	References
Protegrin-1 + Colistin	Dual membrane disruption: PG-1 permeabilises the bacterial outer membrane, enhancing colistin’s binding to LPS and subsequent inner membrane disruption.	MDR Gram-negative bacteria (e.g., *Pseudomonas aeruginosa*, *Acinetobacter baumannii*, *Klebsiella pneumoniae*)	Synergistic reduction in minimum inhibitory concentrations (MICs) and improved bactericidal activity compared to monotherapy.	Morroni et al. [14] Brogden [34]
Protegrin-1 + Rifampicin	Membrane permeabilisation by PG-1 increases intracellular accumulation of rifampicin, which inhibits RNA polymerase and transcription.	MDR Gram-positive bacteria (e.g., methicillin-resistant *Staphylococcus aureus*)	Enhanced bactericidal effect with reduced emergence of resistance, due to improved drug uptake and complementary modes of action.	Hancock et al. [35] Shai [23]
Protegrin-1 + Meropenem	PG-1-induced disruption of the bacterial membrane facilitates the entry of meropenem.	MDR Enterobacteriaceae (including extended-spectrum β-lactamase-producing strains)	Synergistic killing with lower effective doses of meropenem, resulting in enhanced clearance of resistant bacterial populations.	Brogden [34] Hancock et al. [35]
Protegrin-1 + Vancomycin	*Enhanced penetration:* PG-1-mediated membrane disruption facilitates the uptake of vancomycin, which interferes with cell wall synthesis in bacteria that are otherwise resistant due to biofilm formation or thick cell walls.	MDR Gram-positive bacteria, including vancomycin-resistant enterococci (VRE) and MRSA	Combination therapy resulted in significant MIC reductions for vancomycin, synergistic bactericidal activity in vitro, and enhanced therapeutic efficacy in animal infection models.	Morroni et al. [14] Shai [23] Hancock et al. [35] Boman [36]

## Data Availability

All data are in the manuscript.
